# Supplementary material for: Engineering consortia by polymeric microbial swarmbots
Source: Nat Commun. 2022 Jul 5;13:3879. doi: 10.1038/s41467-022-31467-1 (PMC9256712; doi:10.1038/s41467-022-31467-1)
Supplement: Supplementary file 1 — Supplementary Information [file 41467_2022_31467_MOESM1_ESM.pdf]

# Engineering consortia by polymeric microbial swarmbots

Wang *et al.*

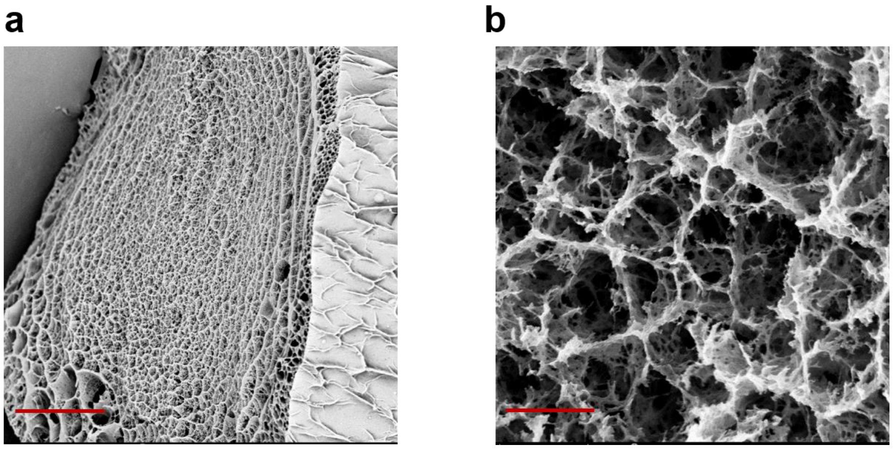


**Supplementary Figure 1. SEM images reflected the porous structure of MSB.** The three-dimensional crosslinked structure can trap the microbes, support their growth and allow the free diffusion of nutrients, signaling molecules, metabolites and proteins. Scale bar = 30 μm (**a**) and 3 μm (**b**). Experiments were repeated independently more than three times with similar results.

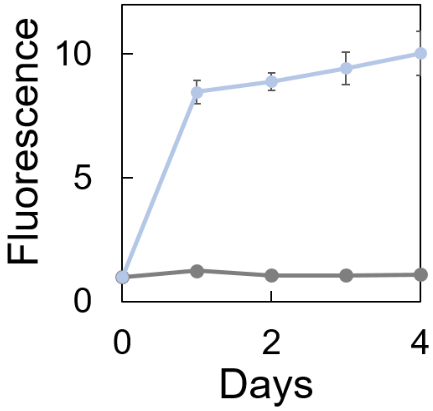


**Supplementary Figure 2. Porous structure of MSB permitted the transport of macromolecules.** We encapsulated dextran-rhodamine (Mw ~ 150kDa) in chitosan capsules and immersed these capsules in PBS. Fluorescence in the surrounding medium was quantified at different time points. Compared with the empty capsules (grave curve), the porous structure permitted the transport of the macromolecules (blue curve). The y-axis is in arbitrary units. Error bars = Standard Deviation (n = 3 biologically independent samples). Source data are provided as a Source Data file.

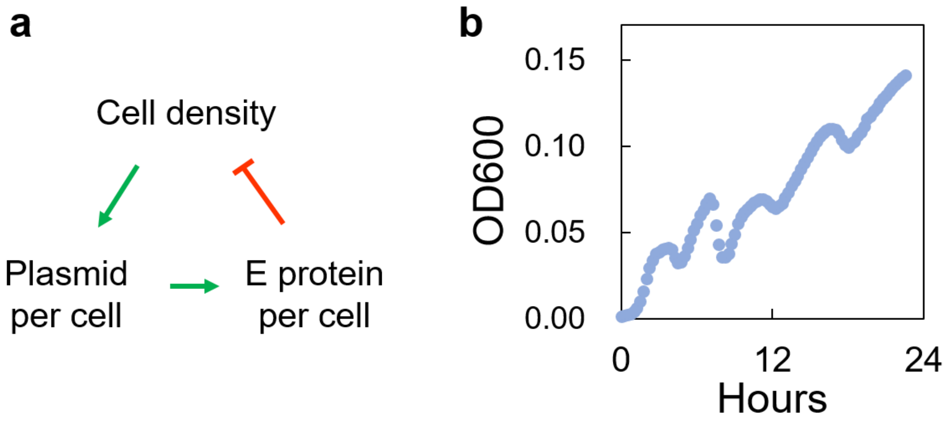


**Supplementary Figure 3. Self-lysis of *E. coli* programmed by the engineered circuit.**

**a.** **Logic of the lysis module.** The core of the ePop circuit is the basal-level expression of a toxin (the E protein from phage phiX174) from a plasmid, whose copy number depends on the bacterial density. Bacterial growth leads to an increase in plasmid copy number, which causes the expression and accumulation of the E protein. The E protein interferes with the cell wall synthesis and causes cell lysis at sufficient high concentration.

**b.** **Experimental measurements of the ePop dynamics.** The circuit generated population-level oscillations in BL21(DE3) cells when cultured in M9 medium at 37$^{o}$C. Source data are provided as a Source Data file.

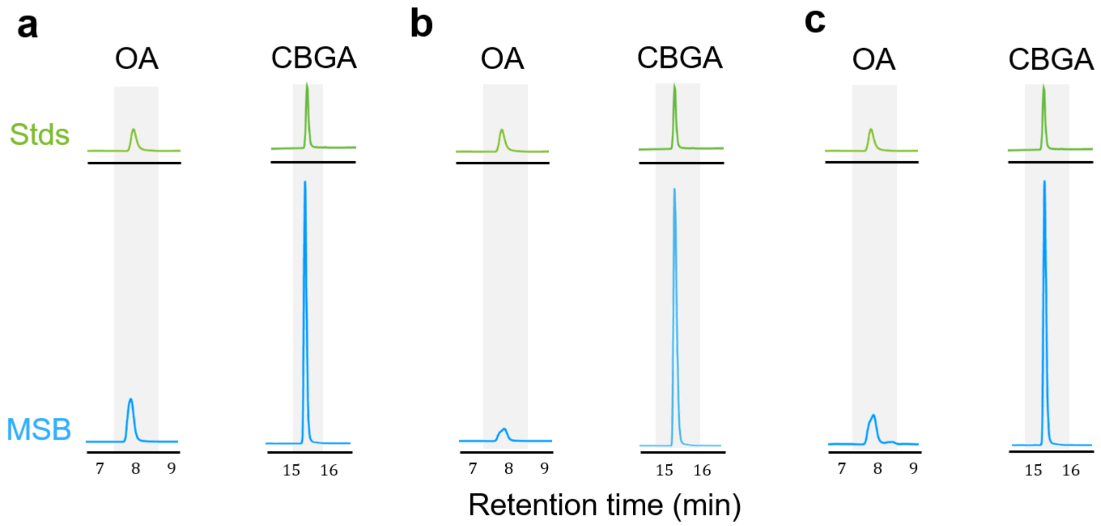


**Supplementary Figure 4. MSB(*S. cerevisiae*) produced CBGA by feeding olivetolic acid (OA).** MSB(*S. cerevisiae*) fed with 1 mM OA were sampled 96 hrs after induction. Extracts were analyzed by HPLC and signals were compared to genuine standards (50 μM for OA and CBGA standard samples). All HPLC chromatograms were selected for the theoretical m/z values (shown in **Supplementary Figure 5**) of the respective compounds of interest. The experiments were repeated three times as shown in **a-c**. Source data are provided as a Source Data file.

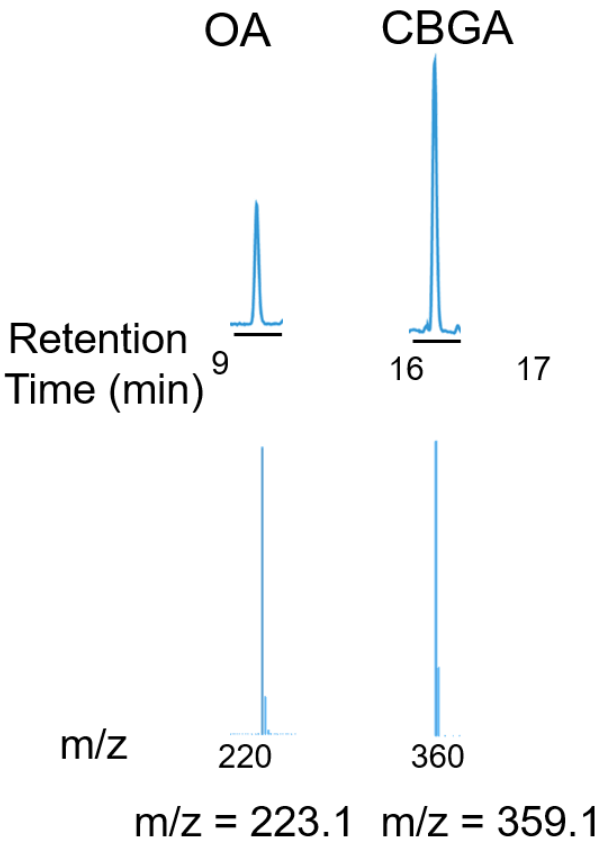


**Supplementary Figure 5. Mass spectra of CBGA produced by MSB(*S. cerevisiae*).** The $m/z_{theoretical}$ value for OA and CBGA is 223.097 and 359.223, respectively. Source data are provided as a Source Data file.

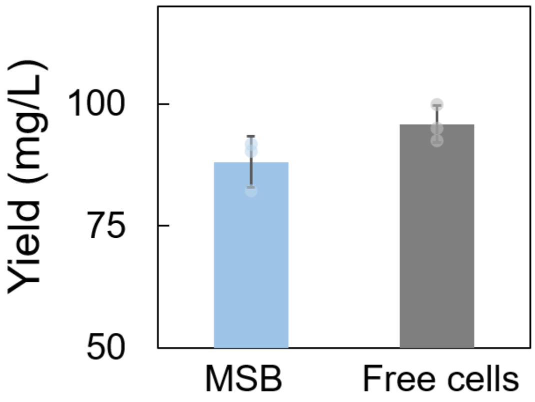


**Supplementary Figure 6. MSB(*S. cerevisiae*) had a similar yield of CBGA compared with the free cells.** Error bars = Standard Deviation (n = 3 biologically independent samples). Source data are provided as a Source Data file.

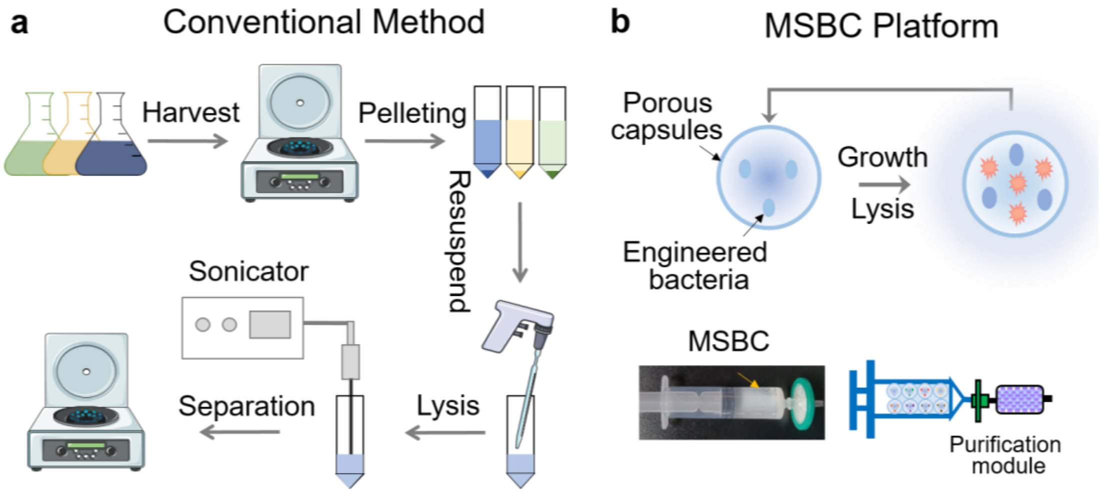


**Supplementary Figure 7. MSBC technology integrates production, lysis and separation into a concise format.**

**a. Conventional method for protein biomanufacturing involves multiple cycles of centrifuging and pipetting.** The lysis procedure of a conventional method typically involves additional equipment, such as centrifuge and sonicator.

**b. MSBC technology does not need access to large equipment or even electricity.** The procedure is continuous and easy to operate.

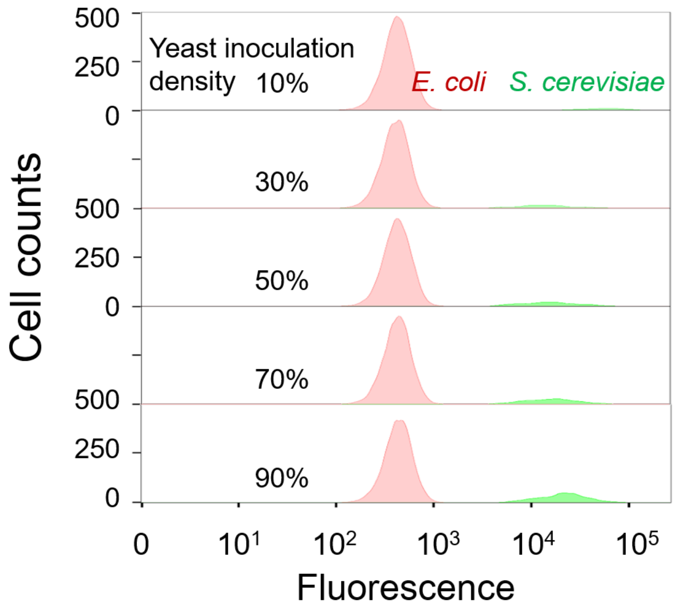


**Supplementary Figure 8. Flow cytometry results reflected that the faster-growing *E. coli* overtook the slower-growing *S. cerevisiae* in a well-mixed consortium.** The areas of the red peak (*E. coli*) and green peak (*S. cerevisiae*) indicated the cell populations of the two species. The x-axis is in arbitrary units.

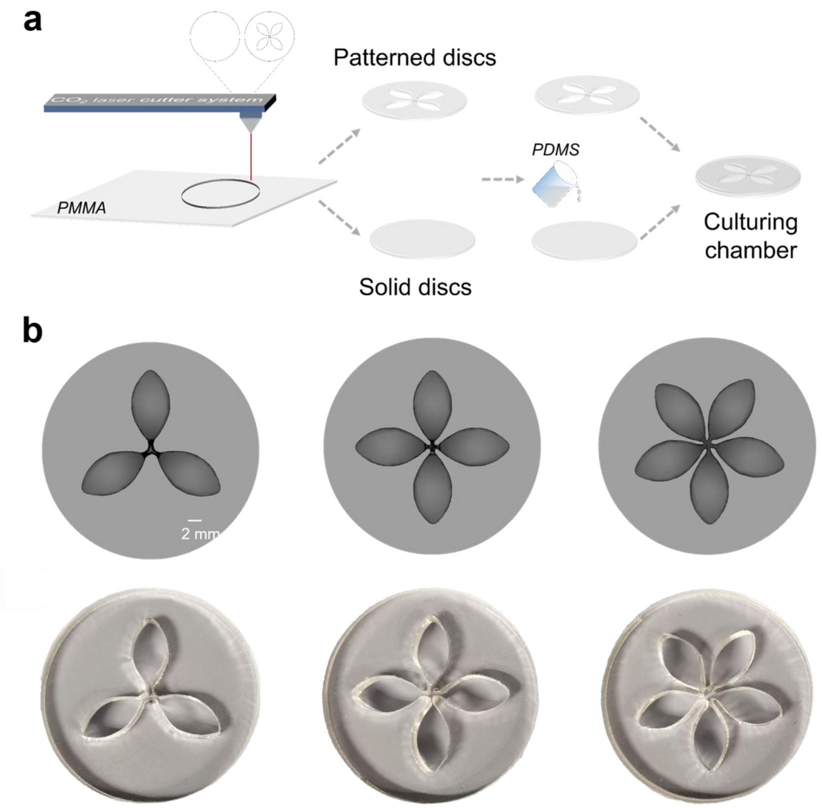


**Supplementary Figure 9. Fabrication of flower-shaped polymethyl methacrylate (PMMA) plates.**

a. **Fabrication of the PMMA plates.** Transparent PMMA (thickness = 5 mm) was cut into bottom discs and top discs (with the flower-shaped structure) by a $CO_2$ laser cutter system. The bottom and top discs were then sealed by polydimethylsiloxane (PDMS) adhesive.

b. **The plates were cut into the desired shape based on the design.** Scale bar = 2 mm.

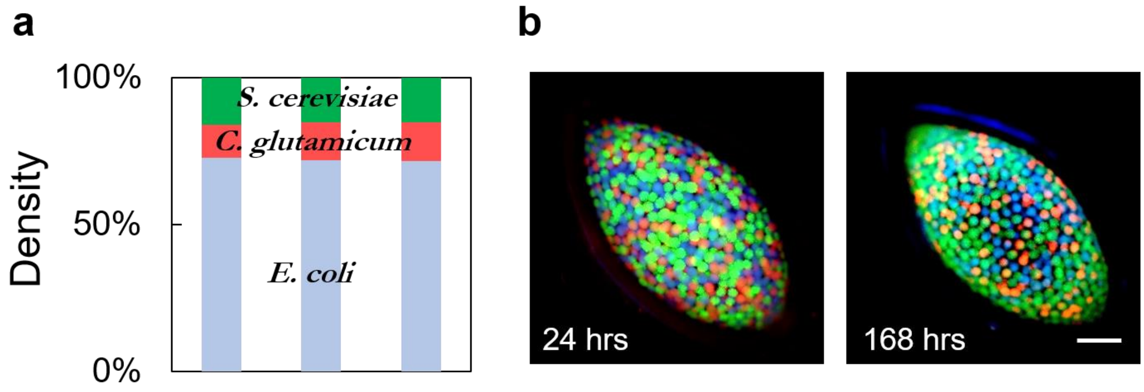


**Supplementary Figure 10. MSBC method can assemble the three-species consortia with precision and durability.**

**a.** **Flow cytometry results reflected that the faster-growing *E. coli* overtook the slower-growing species in a well-mixed consortium.** Homogeneous culture of the *E. coli*, *S. cerevisiae* and *C. glutamicum* (seeding ratio = 1:1:1) led to the domination of only one species (*E. coli*) at the end. Experiments were repeated independently more than three times with similar results. Source data are provided as a Source Data file.

**b.** **MSBC method stabilized the system for over 7 days.** MSBC were assembled by mixing the MSB(*E. coli*), MSB(*S. cerevisiae*) and MSB(*C. glutamicum*), and the system was well maintained even after 7 days, underscoring its stability. Scale bar = 1 mm. Experiments were repeated independently more than three times with similar results.

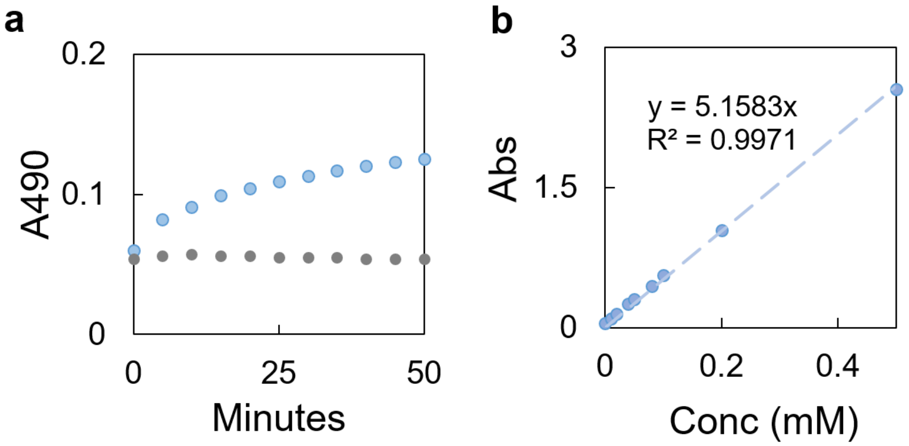


**Supplementary Figure 11. The division of labor product of MSBC had both Bla and rh-PON-1 activity.**

**a. Nitrocefin assay confirmed the Bla activity.** 5 μL of collected supernatant was diluted into 100 μL by PBS and then mixed with the substrate nitrocefin (50 μM). The y-axis showed the absorbance at 490 nm. When the substrate is saturating, the initial slope of the absorbance is proportional to the activity of the Bla (see **Methods**). Compared with control (gray), the DOL product demonstrated the Bla activity (blue).

**b. Standard curve of PNP.** We plotted the concentration of PNP against its absorbance at 405 nm. This curve was used to calculate the PNP generation during the PAR hydrolysis. Source data are provided as a Source Data file.

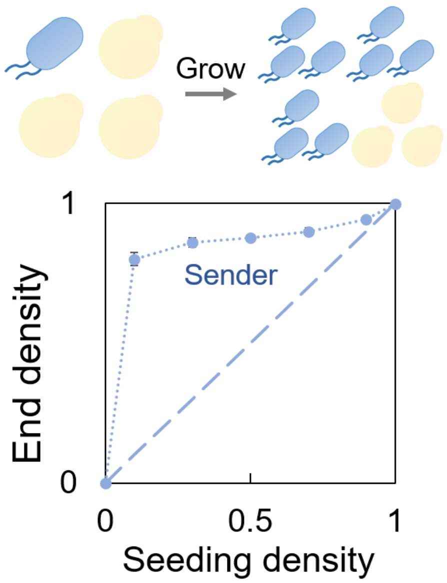


**Supplementary Figure 12. The faster-growing sender cells (*E. coli*) overtook the slower-growing receiver cells (*S. cerevisiae*) in a well-mixed consortium.** Regardless of the seeding ratio, sender cells (*E. coli*) became the dominant in the well-mixed consortia. The dotted and dashed lines showed the actual and ideal composition respectively. Error bars = Standard Deviation (n = 3 biologically independent samples). Source data are provided as a Source Data file.

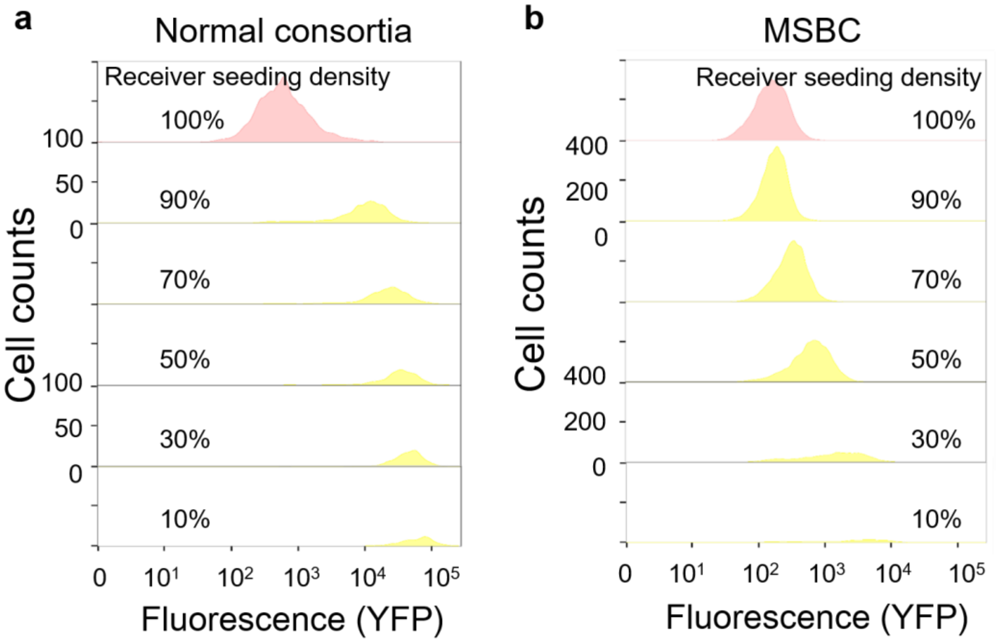


**Supplementary Figure 13. Flow cytometry results confirmed that MSBC platform could tightly control the ratio between the sender cells and receiver cells.**

a. **Flow cytometry results confirmed that simply mixing the free cells could not control the composition of the subpopulations.** The peak area was related to the cell density, while the median of the yellow peak reflected the fluorescence of the receiver. The drastic shift in the fluorescence signal (compared to the control, red peak) was due to the activation of receiver cells by the faster-growing sender cells even when the seeding density of the sender cells equals 10% (**Supplementary Figure 12**). The x-axis is in arbitrary units. Experiments were repeated independently more than three times with similar results.

b. **Flow cytometry results confirmed that the MSBC platform could tightly control the composition of the subpopulations.** The peak area was related to the cell density, while the median of the yellow peak reflected the fluorescence of the receiver. The gradual shift in the density (peak area) as well as the fluorescence (peak median) of the receiver cells suggested the strict population control by MSBC method. The x-axis is in arbitrary units. Experiments were repeated independently more than three times with similar results.

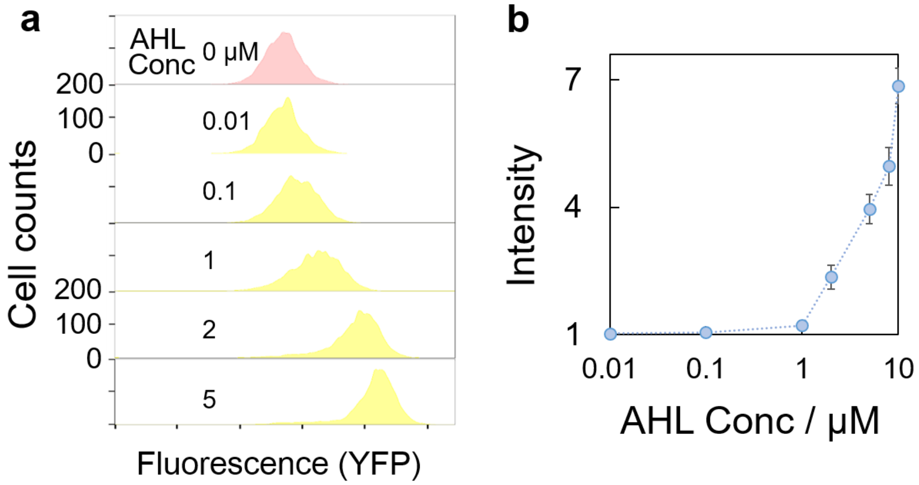


**Supplementary Figure 14. Titrating the receiver cells with AHL caused the gradual increase in the fluorescence intensity.**

a. **Titrating the receiver cells with the signaling molecules led to the gradual increase in the fluorescence (peak median).** The peak area was related to the cell density, while the median of the yellow peak reflected the fluorescence of the receiver. The number in each panel indicated the AHL concentrations. The x-axis is in arbitrary units. Experiments were repeated independently more than three times with similar results.

b. **The trend of signal intensity by titrating was comparable with that of the MSBC platform.** The fluorescence intensity of *S. cerevisiae* (receiver) was quantified through the flow cytometry (the median of the peak) and normalized by the value when AHL = 0.01 μM. The x-axis indicated the concentrations of AHL. Error bars = Standard Deviation (n = 3 biologically independent samples). Source data are provided as a Source Data file.

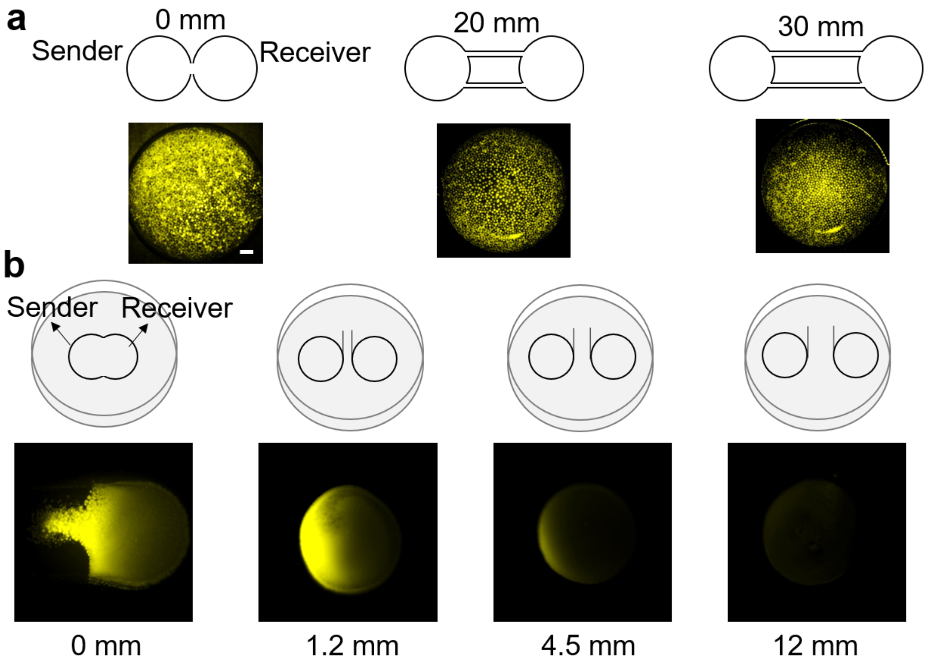


**Supplementary Figure 15. MSBC platform allowed the modulation of signal range in a flexible manner.**

**a. MSBC platform modulated the signal range in a flexible manner.** We fabricated several micro-device with two chambers connected by channels of different lengths. We inoculated the $MSB_{sender}$ and $MSB_{receiver}$ into the chambers separately. Results showed that the cells of $MSB_{receiver}$ were activated at different spatial ranges, and the fluorescence of the $MSB_{receiver}$ was homogeneous. Scale bar = 2 mm.

**b. The expansion caused by the growth of the free cells pushed the sender and receiver cells out of the optimal range of communication.** In the free cells system, the fluorescence of the receiver was not homogeneous since the growth of the sender and receiver cells pushed the cells far apart. Increasing the spatial range of the sender and receiver cells permitted the cells to grow independently without the interference. However, the signal quickly deactivated as shown by the diminished fluorescence of the receiver cells.

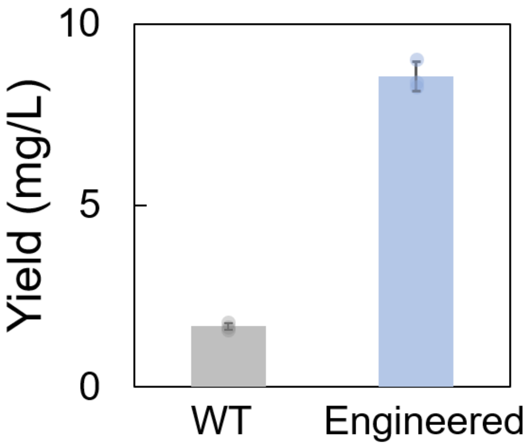


**Supplementary Figure 16. Engineered *S. elongatus* produced sucrose under the sodium chloride stress.** *S. elongatus* were cultured in BG-11 and stressed by supplementing 150 mM NaCl. The yield of sucrose was measured by a sucrose quantification kit (see **Methods**). Error bars = Standard Deviation (n = 3 biologically independent samples). Source data are provided as a Source Data file.

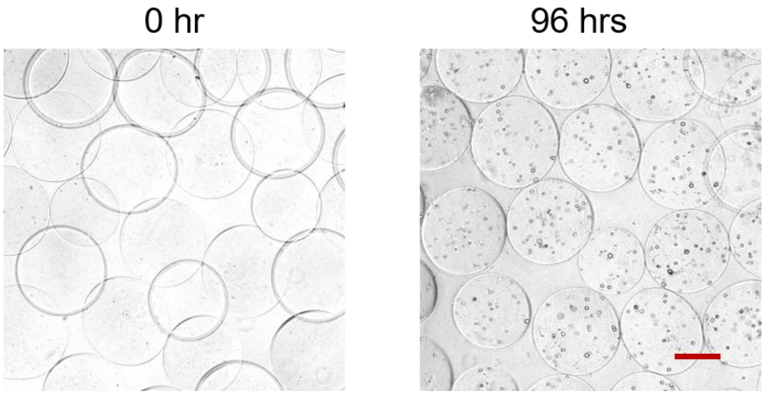


**Supplementary Figure 17. MSB(*E. coli*) grew in CoBG-11 medium supplemented with 0.2% sucrose.** We used *E. coli* (sucrose-utilizing strain) to generate the MSB(*E. coli*). By culturing these MSB in CoBG-11 medium supplemented with 0.2% sucrose, colonies appeared inside the MSB after 96 hrs. Scale bar = 300 μm. Experiments were repeated independently more than three times with similar results.

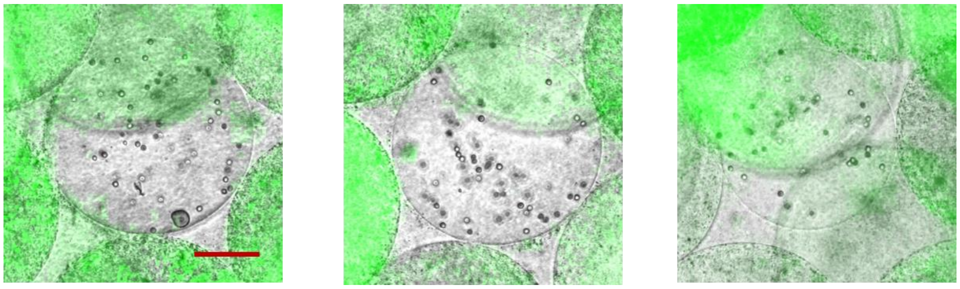

**Supplementary Figure 18. *E. coli* grew inside MSBC in minimal media devoid of any organic carbon source.** The phototrophic MSB(*S. elongatus*) successfully sustained the heterotrophic MSB(*E. coli*) at a high MSB(*S. elongatus*) seeding density (seeding ratio of $MSB_{phototroph}$ and $MSB_{heterotroph}$ equals 50:1). Scale bar = 150 μm. Experiments were repeated independently more than three times with similar results.

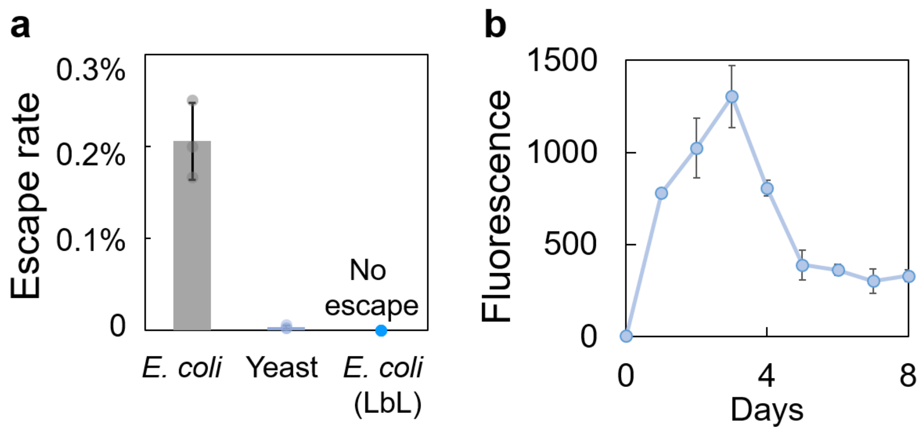


**Supplementary Figure 19. MSBC system can be optimized for separation efficacy and used for long-term.**

a. **The separation efficacy could be modulated by surface modification.** Escape rate is defined as the percentage of the escaped cells in terms of total cell number. It is calculated as dividing the number of the cells in the surrounding medium of MSB by the number of cells in MSB (see **Methods**). After LbL modification, the escape rate of the cells of MSB(*E. coli*) decreased to zero. Error bars = Standard Deviation (n = 3 biologically independent samples).

b. **MSBs were reused for 8 days.** *E. coli* MC4100Z1(ePop/mCherry) cells were encapsulated inside the capsules and cultured in M9 at 37°C. Every 24 hours, the supernatant was harvested and the new medium was added to the MSB. The fluorescence of the supernatant was evaluated by a platereader. The y-axis is in arbitrary units. Error bars = Standard Deviation (n = 3 biologically independent samples). Source data are provided as a Source Data file.

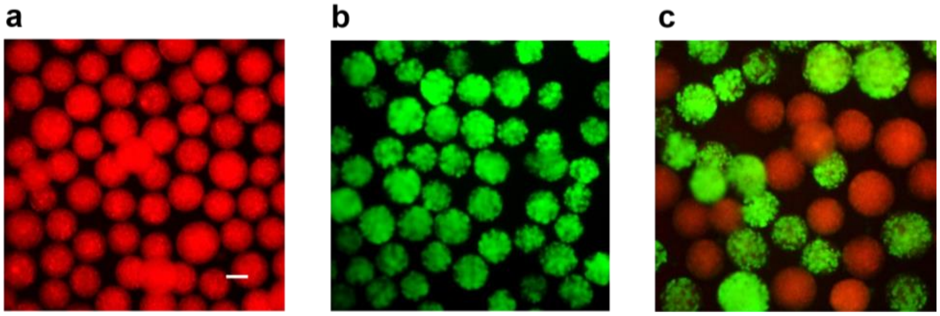


**Supplementary Figure 20. MSBs could be stored for 12 months for on-demand assembly of MSBC.** The MSBs were prepared and stored at -80 $^{o}$C. The MSBs could be used anytime by supplementing the nutrients. **a**, **b** and **c** showed that the MSB(*E. coli*), MSB(*S. cerevisiae*) and MSBC assembled by these two stored MSBs actively grew in the nutrients. Scale bar = 200 μm. Experiments in **a-c** were repeated independently more than three times with similar results.

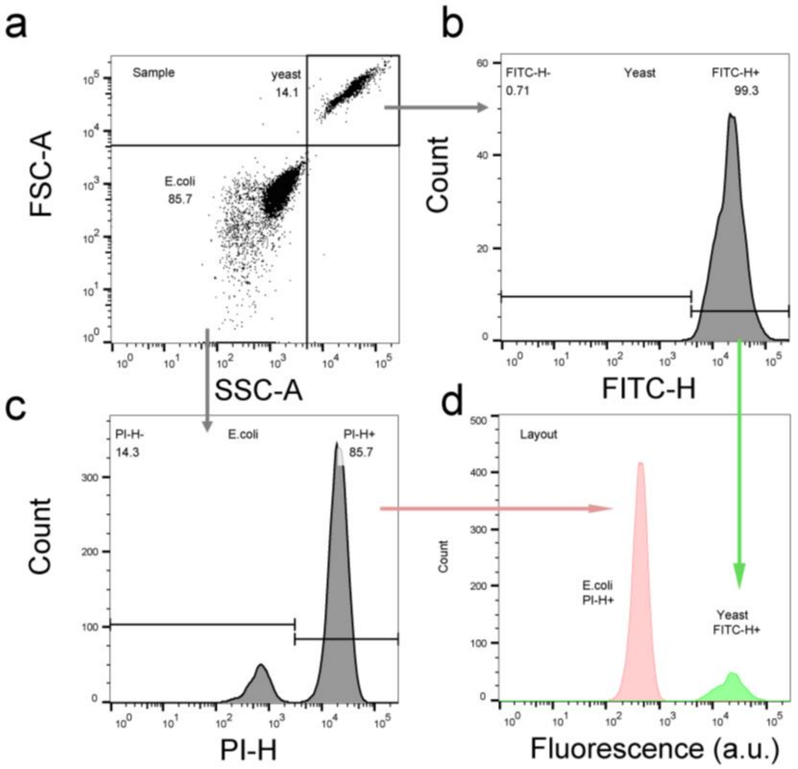


**Supplementary Figure 21. The gating strategy in flow cytometry analysis.** We used FlowJo to analyze the sample data. We selected FSC-A≥5.0kV, SSC-A≥5.0kV and FSC-A≤5.0kV, SSC-A≤5.0kV to distinguish *S. cerevisiae* and *E. coli* (**a**). In the corresponding gates, FITC-H≥4.0kV and PI-H≥3.0kV were further selected to distinguish cells with fluorescent signals (**b** and **c**). Finally, the data processing results were obtained by adjusting the layout (**d**).

**Supplementary Table 1. Protein sequences of the constructs**

| Protein | Amino acid sequences |
|---|---|
| rh-GH | FPTIPLSRLFDNAMLRAHRLHQLAFDTYQEFEEAYIPKEQKYSFLQ NPQTSLCFSESIPTPSNREETQQKSNLELLRISLLLIQSWLEPVQFLR SVFANSLVYGASDSNVYDLLKDLEEGIQTLMGRLEDGSPRTGQIF KQTYSKFDTNSHNDDALLKNYGLLYCFRKDMDKVETFLRIVQCR SVEGSCGF |
| rh-PON1 | MAKLIALTLLGMGLALFRNHQSSYQTRLNALREVQPVELPNCNL VKGIETGSEDLEILPNGLAFISSGLKYPGIKSFNPNSPGKILLMDLNE EDPTVLELGITGSKFDVSSFNPHGISTFTDEDNAMYLLVVNHPDAK STVELFKFQEEEKSLLHLKTIRHKLLPNLNDIVAVGPEHFYGTNDH YFLDPYLQSWEMYLGLAWSYVVYYSPSEVRVVAEGFDFANGINI SPDGKYVYIAELLAHKIHVYEKHANWTLTPLKSLDFNTLVDNISV DPETGDLWVGCHPNGMKIFFYDSENPPASEVLRIQNILTEEPKVTQ VYAENGTVLQGSTVASVYKGKLLIGTVFHKALYCELLE |
| LasI | MIVQIGRREEFDKKLLGEMHKLRAQVFKERKGWDVSVIDEMEID GYDALSPYYMLIQEDTPEAQVFGCWRILDTTGPYMLKNTFPELLH GKEAPCSPHIWELSRFAINSGQKGSLGFSDCTLEAMRALARYSLQ NDIQTLVTVTTVGVEKMMIRAGLDVSRFGPHLKIGIERAVALRIEL NAKTQIALYGGVLVEQRLAVS |
| LasR | ALVDGFLELERSSGKLEWSAILQKMASDLGFSKILFGLLPKDSQDY ENAFIVGNYPAAWREHYDRAGYARVDPTVSHCTQSVLPIFWEPSI YQTRKQHEFFEEASAAGLVYGLTMPLHGARGELGALSLSVEAEN RAEANRFMESVLPTLWMLKDYALQSGAGLAFEHPVSKPVVLTSR EKEVLQWCAIGKTSWEISVICNCSEANVNFHMGNIRRKFGVTSRR VAAIMAVNLGLITL |
| cscB | MALNIPFRNAYYRFASSYSFLFFISWSLWWSLYAIWLKGHLGLTG TELGTLYSVNQFTSILFMMFYGIVQDKLGLKKPLIWCMSFILVLTG PFMIYVYEPLLQSNFSVGLILGALFFGLGYLAGCGLLDSFTEKMAR NFHFEYGTARAWGSFGYAIGAFFAGIFFSISPHINFWLVSLFGAVF MMINMRFKDKDHQCVAADAGGVKKEDFIAVFKDRNFWVFVIFIV GTWSFYNIFDQQLFPVFYSGLFESHDVGTRLYGYLNSFQVVLEAL CMAIIPFFVNRVGPKNALLIGVVIMALRILSCALFVNPWIISLVKLL HAIEVPLCVISVFKYSVANFDKRLSSTIFLIGFQIASSLGIVLLSTPTG ILFDHAGYQTVFFAISGIVCLMLLFGIFFLSKKREQIVMETPVPSAI |
