# Supplementary material for: Engineering consortia by polymeric microbial swarmbots
Source: Nat Commun. 2022 Jul 5;13:3879. doi: 10.1038/s41467-022-31467-1 (PMC9256712; doi:10.1038/s41467-022-31467-1)
Supplement: Supplementary file 3 — Description of Additional Supplementary Files [file 41467_2022_31467_MOESM3_ESM.pdf]

File name: Supplementary Movie 1

Description: The co-culture of *E. coli* and *S. cerevisiae* by MSBC platform. The MSBC were cultured at the room temperature (~22 ºC).
