# Supplementary material for: Engineering consortia by polymeric microbial swarmbots
Source: Nat Commun. 2022 Jul 5;13:3879. doi: 10.1038/s41467-022-31467-1 (PMC9256712; doi:10.1038/s41467-022-31467-1)
Supplement: Supplementary file 5 — Reporting Summary [file 41467_2022_31467_MOESM5_ESM.pdf]

nature portfolio

Corresponding author(s): ZHUOJUN DAI

Last updated by author(s): Jun 10, 2022

Reporting Summary

Nature Portfolio wishes to improve the reproducibility of the work that we publish. This form provides structure for consistency and transparency in reporting. For further information on Nature Portfolio policies, see our Editorial Policies and the Editorial Policy Checklist.

Statistics

For all statistical analyses, confirm that the following items are present in the figure legend, table legend, main text, or Methods section.

n/a	Confirmed	
☐	☒	The exact sample size (*n*) for each experimental group/condition, given as a discrete number and unit of measurement
☐	☒	A statement on whether measurements were taken from distinct samples or whether the same sample was measured repeatedly
☒	☐	The statistical test(s) used AND whether they are one- or two-sided<br>*Only common tests should be described solely by name; describe more complex techniques in the Methods section.*
☒	☐	A description of all covariates tested
☒	☐	A description of any assumptions or corrections, such as tests of normality and adjustment for multiple comparisons
☐	☒	A full description of the statistical parameters including central tendency (e.g. means) or other basic estimates (e.g. regression coefficient) AND variation (e.g. standard deviation) or associated estimates of uncertainty (e.g. confidence intervals)
☒	☐	For null hypothesis testing, the test statistic (e.g. *F*, *t*, *r*) with confidence intervals, effect sizes, degrees of freedom and *P* value noted<br>*Give P values as exact values whenever suitable.*
☒	☐	For Bayesian analysis, information on the choice of priors and Markov chain Monte Carlo settings
☒	☐	For hierarchical and complex designs, identification of the appropriate level for tests and full reporting of outcomes
☒	☐	Estimates of effect sizes (e.g. Cohen's *d*, Pearson's *r*), indicating how they were calculated

Our web collection on statistics for biologists contains articles on many of the points above.

Software and code

Policy information about availability of computer code

Data collection	The i-control 2.0 software was used for collecting absorbance and fluorescence data with the Infinite 200pro multimode reader (TECAN). Gen5 CHS software (version 3.08) was used for collecting absorbance and fluorescence data with Biotek Synergy H1 and Biotek Epoch 2. Image lab 6.0 software (version 6.0) was used for collecting SDS-PAGE with the Molecular Imager Gel Doc XR+ system (BIO-RAD). BD FACSDiva software (version 8.0.1.1) was used for collecting flow cytometry data with BD FACSCelestaTM. Agilent Openlab software was used for collecting HPLC data with Agilent 1260 Infinity II. Agilent MassHunter software was used for collecting LC/MS data with Agilent 6470B LC/TQ. MATLAB R2018b was used for collecting microscopic and fluorescence images with Olympus MVX10. NIS-Elements software (version 11.0.0.28844) was used for collecting microscopic and fluorescence images with Nikon-Ti2. The phenom UI software was used for collecting SEM data with Phenom Pharos Desktop SEM.
Data analysis	Imaging data analysis was performed using Image J 152-win-java8. FlowJo (version 10.6.2) was used for analyzing flow cytometry data. Agilent Openlab Data Analysis software was used for analyzing HPLC data. Agilent MassHunter Qualitative Analysis software was used for analyzing LC/MS data. Microsoft Excel (2013) was used for plot generation and statistical analysis.

For manuscripts utilizing custom algorithms or software that are central to the research but not yet described in published literature, software must be made available to editors and reviewers. We strongly encourage code deposition in a community repository (e.g. GitHub). See the Nature Portfolio guidelines for submitting code & software for further information.

Data

Policy information about availability of data

All manuscripts must include a data availability statement. This statement should provide the following information, where applicable:

- Accession codes, unique identifiers, or web links for publicly available datasets
- A description of any restrictions on data availability
- For clinical datasets or third party data, please ensure that the statement adheres to our policy

The authors declare that the source data processed for figures generation in this study are available within the paper and its supplementary files. Any additional information is available upon request.

Field-specific reporting

Please select the one below that is the best fit for your research. If you are not sure, read the appropriate sections before making your selection.

☒ Life sciences ☐ Behavioural & social sciences ☐ Ecological, evolutionary & environmental sciences

For a reference copy of the document with all sections, see nature.com/documents/nr-reporting-summary-flat.pdf

Life sciences study design

All studies must disclose on these points even when the disclosure is negative.

Sample size	The experiments (when replicates were shown) were performed with 3 biologically independent samples. The sample sizes were chosen based on the scale of the project and consistency with other similar published studies (https://doi.org/10.1038/s41467-020-14371-4). No sample size calculation was performed. Our results suggested that the sample sizes we chose were sufficient to test the theoretical predictions.
Data exclusions	No data were excluded.
Replication	Each experiment was repeated at least three times with similar results, suggesting the robustness of our conclusions.
Randomization	For experiments that need overnight culture, clones were picked randomly. Randomization in sample allocation was not necessary because experiments provided consistent quantitative results (see Replication). Covariates/uncontrollable conditions of the experiment did not affect the results, since the appropriate controls were always included in assays to avoid bias.
Blinding	Investigators were not blinded. Knowledge of a samples identity did not affect the experimental conclusion since the data were quantitative and included appropriate controls.

Reporting for specific materials, systems and methods

We require information from authors about some types of materials, experimental systems and methods used in many studies. Here, indicate whether each material, system or method listed is relevant to your study. If you are not sure if a list item applies to your research, read the appropriate section before selecting a response.

Materials & experimental systems

n/a	Involved in the study	
☒	☐	Antibodies
☒	☐	Eukaryotic cell lines
☒	☐	Palaeontology and archaeology
☒	☐	Animals and other organisms
☒	☐	Human research participants
☒	☐	Clinical data
☒	☐	Dual use research of concern

Methods

n/a	Involved in the study	
☒	☐	ChIP-seq
☐	☒	Flow cytometry
☒	☐	MRI-based neuroimaging

Flow Cytometry

Plots

Confirm that:

- [x] The axis labels state the marker and fluorochrome used (e.g. CD4-FITC).
- [x] The axis scales are clearly visible. Include numbers along axes only for bottom left plot of group (a 'group' is an analysis of identical markers).
- [x] All plots are contour plots with outliers or pseudocolor plots.
- [x] A numerical value for number of cells or percentage (with statistics) is provided.

Methodology

Sample preparation	S. cerevisiae, E. coli and C. glutamicum cells were diluted to appropriate density with PBS solution.
Instrument	BD FACSCelestaTM running based on BD FACSDIVA software.
Software	FACS Diva sofeware (version 8.0.1.1) for collection; FlowJo (version 10.6.2) for data analysis.
Cell population abundance	Typical samples contained 10,000 or more cells.
Gating strategy	S. cerevisiae were selected with FSC-A≥5.0 kV, SSC-A≥5.0 kV and then screen out the cells that FITC-H≥4.0 kV. E. coli were selected with FSC-A≤5.0 kV, SSC-A≤5.0 kV and then screen out the cells that PI-H≥3.0 kV. C. glutamicum were selected with Pacific Blue-A ≥540 V. We have provided a figure (Supplementary Figure 21) to elabroate the gating strategy.

- [x] Tick this box to confirm that a figure exemplifying the gating strategy is provided in the Supplementary Information.
